# Omicron BA.2.75 variant is efficiently neutralised following BA.1 and BA.5 breakthrough infection in vaccinated individuals, Israel, June to September 2022 

**DOI:** 10.2807/1560-7917.ES.2022.27.44.2200785

**Published:** 2022-11-03

**Authors:** Nofar Atari, Limor Kliker, Neta Zuckerman, Bayan Abd Elkader, Yael Weiss-Ottolenghi, Ella Mendelson, Yitshak Kreiss, Gili Regev-Yochay, Michal Mandelboim

**Affiliations:** 1 Central Virology Laboratory, Public Health Services, Ministry of Health and Sheba Medical Center, Tel-Hashomer, Israel; 2 Sackler Faculty of Medicine, Tel-Aviv University, Tel Aviv, Israel; 3 Sheba Medical Center, Tel-Hashomer, Israel

**Keywords:** SARS-CoV-2, Omicron variants, neutralising antibodies

## Abstract

We evaluated neutralising antibody titres against wild type (WT) SARS-CoV-2 and four Omicron variants (BA.1, BA.2, BA.5 and BA.2.75) in fully vaccinated (three doses of Comirnaty vaccine) healthcare workers (HCW) in Israel who had breakthrough BA.1/BA5 infections. Omicron breakthrough infections in vaccinated individuals resulted in increased neutralising antibodies against the WT and Omicron variants compared with vaccinated uninfected HCW. HCW who recovered from BA.1 or BA.5 infections showed similar neutralising antibodies levels against BA.2.75.

The severe acute respiratory syndrome coronavirus 2 (SARS-CoV-2) Omicron (Phylogenetic Assignment of Named Global Outbreak (Pango) lineage designation: B.1.1.529) variant BA.1, was first detected in November 2021 and rapidly spread around the globe. Multiple lineages of the Omicron variants have emerged; BA.2, BA.5 and BA.2.75, which demonstrated high rates of mutation at the spike protein and have the highest transmissibility among the previous SARS-CoV-2 variants. The high genetic variability gives the variants the ability to evade antibodies and causes re-infections [1]. There is still very limited information about the neutralisation efficiency of vaccinated individuals against BA.2.75. To address this, we used here the same methods that were used previously, to elucidate the vaccine effectiveness against BA.5 [2]. 

## The BA.5 and BA.2.75 variants in Israel

**Figure 1:**
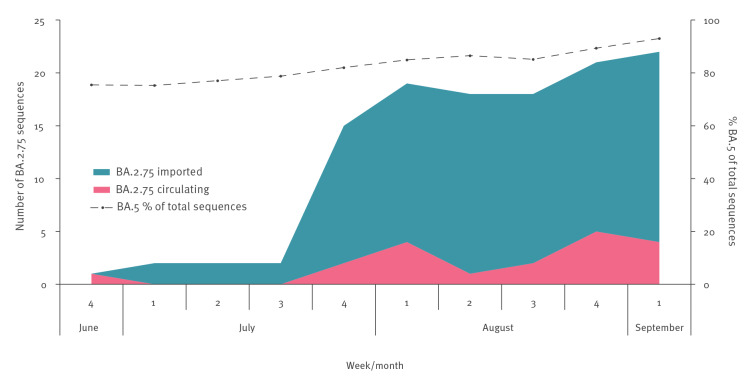
SARS-CoV-2 Omicron BA.2.75 variant, circulating and imported, and BA.5 variant circulating, Israel, June–September 2022

The frequency of SARS-CoV-2 variants BA.5 and BA.2.75 in Israel was monitored by whole genome sequencing of SARS-CoV-2-positive samples (Figure 1). BA.2.75 was first detected in Israel in June 2022 in an individual arriving from India. While importation of BA.2.75 into Israel has increased, in concordance with the global increase in BA.2.75 [3], its circulation in Israel has not increased (Figure 1). This may be due to the dominance of BA.5 at the time of BA.2.75 detection and spread in Israel, ranging from 75% of the total number of sequenced samples in Israel in the beginning of June to 95% in the beginning of September.

SARS-CoV-2: severe acute respiratory syndrome coronavirus 2.

## Neutralisation efficiency against SARS-CoV-2 BA.2.75 variant

Using the same study setup and methodology as described earlier [2], we evaluated neutralising antibody titres against wild-type (WT) sub-lineage B.1.1.50 and the four Omicron variants (BA.1, BA.2, BA.5 and BA.2.75) in 55 healthcare workers (HCW) who were monitored by serological follow-up in Israel at different time points between the start of the pandemic in January 2020 and September 2022. We obtained sera from three HCW cohorts. The first cohort included in the analysis were 14 HCW who had received three doses of the Comirnaty vaccine (BNT162b2 mRNA, Pfizer/BioNTech) and were SARS-CoV-2-naïve (Column 1 in the Table). Sera were obtained from this group 5 months after the third vaccine dose. The second cohort included 15 previously uninfected HCW who had received three vaccine doses and had a breakthrough infection with BA.1 (Column 2 in the Table). Sera were obtained from this group 5 months after the third vaccine dose and 2 weeks after infection. The third cohort included 26 HCW who had received three vaccine doses and had a breakthrough infection with BA.5. Sera were obtained 3 weeks after infection (on average 10 months after the third vaccine dose, Column 3 in the Table). Demographic data and vaccination information of all HCW participating in this study are provided in the Table.

**Table:** Details of study participants, SARS-CoV-2-neutralising antibodies after vaccination, Israel, June–September 2022 (n = 55)

	HCW who received third vaccination	HCW infected with BA.1 after third vaccination	HCW infected with BA.5 after third vaccination
Number of participants	14	15	26
Age range in years (median)	33–73 (57)	28–68 (48)	37–67 (53)
Average age in years	54	48	52
Males	7	4	2
Females	7	11	24
Days from third vaccination to positive PCR results (average)	143	135	311
Days after positive PCR result (average)	NA	15	21

HCW: healthcare workers; NA: not applicable; SARS-CoV-2: severe acute respiratory syndrome coronavirus 2.

The study included HCW who had received three doses of the Comirnaty vaccine and recovered from SARS-CoV-2 BA.1/BA.5 breakthrough infection.

We obtained whole genome sequenced from cultured virus samples of SARS-CoV-2-positive individuals as previously described [2] to identify the WT (hCoV19/Israel/CVL-45526-ngs/2020), Omicron BA.1 (hCoV-19/Israel/CVL-n49814/2021), Omicron BA.2 (hCoV-19/Israel/CVL-n51046/2022), Omicron BA.5 (hCoV-19/Israel/CVL-n51658/2022) and Omicron, BA.2.75 (hCoV-19/Israel/CVL-n52118/2022). Neutralisation assays were performed as described before [2].

**Figure 2:**
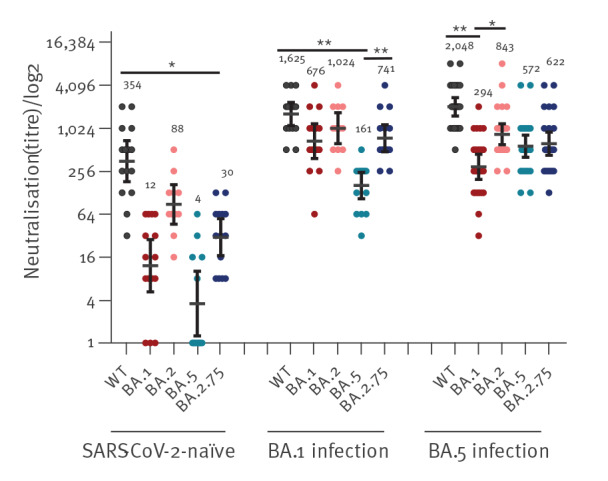
Neutralisation efficiency against wild-type SARS-CoV-2 and the Omicron variants BA.1, BA.2, BA.5 and BA.2.75 in vaccinated healthcare workers, Israel, June–September 2022 (n = 55)

HCW: healthcare workers; SARS-CoV-2: severe acute respiratory syndrome coronavirus 2; WT: wild type.

The neutralisation efficiency in HCW who were infected with BA.1/BA.5 and had previously been vaccinated with three doses of Comirnaty vaccine was significantly higher for all of Omicron variants (unpaired T-test, p value > 0.0008) than in vaccinated but SARS-CoV-2-naïve HCW (Figure 2). The statistical analysis was done in GraphPad prism 9.1.0 (GraphPad Software, San Diego, United States). Additional details on GMT values are provided in Supplementary Table S1.

Serum samples were obtained from 14 HCW who had received three doses of the Comirnaty vaccine (SARS-CoV-2 naïve), 15 HCW who had received three doses of the Comirnaty vaccine and were infected with BA.1 and 26 HCW who had received three doses of the Comirnaty vaccine and were infected with BA.5. Samples were tested with a microneutralisation against WT SARS-CoV-2 and the Omicron variants BA.1, BA.2, BA.5 and BA.2.75. Geometric mean titres (horizontal lines) with 95% confidence intervals (I bars) are presented, as well as the geometric mean titre value at the top of each column. Dots indicate individual serum samples.

* p value < 0.05.

** p value < 0.005.

Sera from HCW who were infected with BA.1 and were previously vaccinated neutralised all Omicron subvariants at a similar efficiency, except for BA.5 which was significantly lower; neutralisation efficiency against BA.1 (geometric mean titre (GMT) = 675.5; 95% CI: 386.6–1,181) was similar to that against BA.2.75 (GMT = 741; 95% CI: 481.1–1,141) (Figure 2). For all GMT values of the study participants please see the additional information in Supplementary Table S1. Moreover, neutralisation efficiency against BA.5 in HCW recovered after infection with BA.5 showed similar but inversely proportional results to those seen in HCW who recovered from BA.1: the levels of neutralisation were lower against BA.1 but similar in BA.5 and BA.2.75 with GMT values of 294.1 (95% CI: 196.2–440.7), 572.1 (95% CI: 400.3–817.5) and 622 (95% CI: 427.6–903.7), respectively.

## Discussion

The SARS-CoV-2 Omicron variant was discovered in November 2021 in South Africa. Subsequently, several lineages of Omicron were identified, named BA.2, BA.4 and BA.5, which caused several waves of infection and are of interest due to their genetic diversity [4]. These variants contain more than 30 mutations in their spike protein region, compared with both the Alpha (B.1.1.7) and the Delta (B.1.617.2) variants [5,6]. A new BA.2 sub-lineage, BA.2.75, was detected in India during June 2022 and is the first SARS-CoV-2 variant of second generation that is reported to spread outside its original region [3]. BA.2.75 appears to be more transmissible than other Omicron variants which results in compromised vaccine efficiency [7,8]. In addition to the existing mutations reported in the other Omicron variants [5,6], BA.2.75 contains nine novel mutations in the spike protein. Two of them, G446S and R493Q, are currently being studied for their pathogenic attributes. The R493Q mutation increases the ability of the spike protein to bind to the human ACE2 receptor, while the G446S mutation affects both immune evasion and ACE2 binding [8-10]. 

In this study, we analysed the neutralisation efficiency of the Comirnaty vaccine against wild-type SARS-CoV-2 and the four Omicron variants BA.1, BA.2, BA.5 and BA.2.75 in vaccinated individuals and those who recovered from BA.1 or BA.5 infection. As expected, neutralisation efficiency against all Omicron variants was significantly higher in individuals who recovered from BA.1 or BA.5 infection compared with vaccinated SARS-CoV-2-naïve individuals. Vaccinated individuals infected with BA.1 had similar neutralisation against BA.1 and BA.2.75, but lower neutralisation of BA.5. Conversely, if an individual was exposed BA.5, the results show similar neutralisation against BA.5 and BA.2.75, but significantly less against in BA.1. In addition, another study showed that individuals who were vaccinated with two doses of BBIBP-CorV (Sinopharm) and received a booster with the BBIBP-CorV or Zifivax (Anhui Zhifei Longcom) vaccine had lower antibody titres against BA4 or BA5 than against BA2.75 [11].

## Conclusion

Our data show that there is good cross-reactivity of SARS-CoV-2 BA.1 and BA.5 with BA.2.75. The BA.2.75 variant escapes immune response to a lesser degree than BA.1 and BA.5, and vaccinated individuals recovered from infection with an Omicron variant will probably be protected from BA.2.75 infection. This may explain why BA.2.75 did not spread in countries were BA.5 was dominant. 

## Supplementary Data

142000 Supplement
