# Supplementary material for: Omicron BA.2.75 variant is efficiently neutralised following BA.1 and BA.5 breakthrough infection in vaccinated individuals, Israel, June to September 2022
Source: Euro Surveill. 2022 Nov 3;27(44):2200785. doi: 10.2807/1560-7917.ES.2022.27.44.2200785 (PMC9635020; doi:10.2807/1560-7917.ES.2022.27.44.2200785)
Supplement: Supplement [file 22-00785_MANDELBOIM_Supplement.pdf]

Supplementary Appendix

This supplementary material is hosted by *Eurosurveillance* as supporting information alongside the article "Omicron BA.2.75 variant is efficiently neutralised following BA.1 and BA.5 breakthrough infection in vaccinated individuals, Israel, June to September 2022", on behalf of the authors, who remain responsible for the accuracy and appropriateness of the content. The same standards for ethics, copyright, attributions and permissions as for the article apply. Supplements are not edited by *Eurosurveillance* and the journal is not responsible for the maintenance of any links or email addresses provided therein.

Supplementary Table 1: GMT and CI related to study participants included in this report

		3rd dose before infection	3rd dose after infection with BA.1	3rd dose after infection with BA.5
Microneutraliztion assay WT	Geometrical mean	353.8	1625	2048
	Upper and lower 95% CI	182.3-686.3	1118-2364	1521-2758
Microneutraliztion assay Omicron BA.1	Geometrical mean	12.3	675.5	294.1
	Upper and lower 95% CI	5.217-28.19	386.6-1181	196.2-440.7
Microneutraliztion assay Omicron BA.2	Geometrical mean	87.7	1024	843.4
	Upper and lower 95% CI	46.37-165.9	623.1-1683	602.9-1180
Microneutraliztion assay Omicron BA.5	Geometrical mean	3.564	161.3	572.1
	Upper and lower 95% CI	1.256-10.11	105.2-247.2	400.3-817.5
Microneutraliztion assay Omicron BA.2.75	Geometrical mean	30.45	741.0	621.7
	Upper and lower 95% CI	16.76-55.33	481.1-1141	427.6-903.7
